# The single supervisory mechanism and the European framework for the enforcement of competition law: a comparison between two models for economic governance in the EU

**DOI:** 10.1057/s41261-022-00197-1

**Published:** 2022-05-25

**Authors:** Filippo Annunziata, Mariateresa Maggiolino

**Affiliations:** 1 Financial Markets and Banking Law, Banking Law, Bocconi University, Milan, Italy; 2 European Banking Institute, Frankfurt, Germany; 3 Co-Director Unit Rules BAFFI-CAREFIN Research Centre, Università degli Studi di, Milano, Italy; 4 Fellow BAFFI-CAREFIN Research Centre, Bocconi University, Milan, Italy

**Keywords:** ECB, European commission, ECN, SSM, Single supervisory mechanism, Antitrust

## Abstract

This paper proposes to develop a parallel reading of the banking supervision system and the framework for the enforcement of competition law, with the purpose of setting out points of convergence and divergence between the two, analyzing their institutional architecture and various issues pertaining to the relevant, applicable law. This approach might prove fruitful for a better understanding of each of the two systems, and for filling the gaps left open and unresolved by legislative texts. It might also offer some contribution in view of a more general analysis of the key-issues that those who design the systems meant to govern the European economy should consider.

## Introduction

Regulation and supervision of the financial system, and competition law enforcement are two of the most pivotal domains of the European Union’s economic policies: in this context, EU and national institutions have to combine and coordinate their efforts to pursue specific goals and exercise their influence. This paper aims at comparing these distinct frameworks, without the ambition of drawing out a winner from said juxtaposition, but with the goal of identifying the analogous issues the frameworks face, and the parallel solutions they adopt. In other words, by discussing the two systems, the paper develops an analysis that goes beyond law in practice. We begin by singling out the variables with which whoever is interested in designing a legal framework for governing the EU economy must deal. In Paragraph 2, we provide a brief historical introduction of the two legal frameworks that regulate the banking sector and enforce EU competition law. Paragraph 3 serves as an in-depth explanation of what makes the frameworks inherently different. Focusing on their structure and functioning, and despite their evident distinctions, we further argue that they can still be fruitfully compared. In Paragraph 4, we discuss, by focusing on the competences of the institutions involved in the two systems, how each model takes into account the crucial dynamics of interaction between authorities at the EU and national level. In the same vein, Paragraph 5 points out how the two frameworks grants a leadership role to EU Institutions, and Paragraph 6 conceptualizes these systems in relation to the analytical criteria of competences, tasks and uniformity. Paragraph 7 provides closure with an analysis of the legal tools common to both systems. Finally, Paragraph 8 concludes.

## A brief history of the EU banking supervision and competition law enforcement frameworks

Regulation and supervision of the financial system, and competition law enforcement are two of the main areas of EU economic policies, where EU and national institutions have to combine and coordinate their efforts to pursue specific goals and to exercise their influence. While the desire to ensure adequate competition within the Single Market via antitrust rules traces back to the Treaty of Rome of 1957 and to the First Merger Regulation of 1989, financial markets came later into the Agenda of EU institutions, with rules taking their initial shape in the early ‘80 s and, from there on, facing a constant process of transformation and broadening of scope.

With regard to the financial system, the recent reforms introduced within the EU after the global financial crisis of 2007/2008 and the subsequent sovereign debt crisis, have redesigned the scope and contents of most of the European legislation in the field of banking and finance. One of the cornerstones of these reforms was the introduction of the Single Supervisory Mechanism (“SSM”), in the wider context of the European Banking Union project, established in 2013 and in operation since 2014. In a relatively short time span, the SSM has radically reshaped the face of supervision over credit institutions within the Union (and especially in the Euro area),^1^ by integrating national supervision systems and the related National Competent Authorities (“NCAs”) under both the so-called Single Rulebook^2^ and the *aegis* of the European Central Bank ("ECB"). For sure, the SSM has wide implications, including those that touch upon structural issues of European law and its general principles. The questions that arise in the context of the SSM with regard to the principles of proportionality and subsidiarity, the protection of fundamental rights under Union law, the position of European authorities and institutions, the exercise of administrative discretion, for instance, generate strong tensions, while simultaneously confirm the natural tendency of EU law to innovate, and to introduce patterns that often force the reinterpretation of traditional legal categories.^3^

For what matters most here, the SSM is relevant because it designates its “own” way of framing the operations of the many and different EU and national institutions that, by following EU and national laws, must perform banking supervision at both EU and national levels. A similar design can be found in the field of competition and, in particular, in relation to the combined working of the EU and national authorities that are in charge of enforcing competition law at the EU and national levels. For example, some of the rules and parameters governing the allocation of competences between the ECB and the NCAs can find functional equivalents in the rules and criteria according to which the European Commission (“EC”) and the National Competition Authorities (the “Competition NCAs”)^4^ divide their competences in the enforcement of merger control, on the one hand, and in the application of Articles 101 and 102 of the Treaty on the Functioning of the European Union (“TFEU”), on the other.

In particular, pursuant to Regulation 139/2004,^5^ a clear-cut set of rules governs the coordinated enforcement of European and national laws on merger control, by distinguishing between concentrations that deserve to be treated under EU law by the EC and operations that, on the contrary, may be processed by Competition NCAs applying national laws. Therefore, no network of competition authorities is entrusted with the enforcement of the same piece of merger law, although—in allocating cases—the EC works in a constant liaison with the competent authorities of the Member States and cases of deferral are possible. Differently, due to Regulation 1/2003,^6^ today the enforcement of the same legal provisions, i.e., Articles 101 and 102 of the TFEU, is in the hands of a network of agencies, the so-called European Competition Network (“ECN”), where the EC, though playing a leading role, stands together with Competition NCAs in prosecuting anticompetitive agreements and abuses of dominance. Indeed, since 2004, by acting within the ECN, the EC and Competition NCAs have adopted over 1000 decisions, investigating a broad range of cases in all sectors of the economy. In particular, from 2004 until 2014, more than 85% of all the decisions that applied EU antitrust rules were taken by Competition NCAs.^7^

Against this backdrop, this paper proposes to develop a parallel reading of the systems governing banking supervision and the competition law enforcement, with the purpose of setting out points of convergence and divergence between the two, analyzing their institutional framework and various issues pertaining to the relevant, applicable law.^8^ Several other important aspects—for example, the review of legal acts, judicial protection and enforcement—will, in the interest of briefness, only momentarily be touched upon, though the Authors may wish to extend their research in the future and to include these other topics in possible upcoming publications. This approach might prove fruitful for a better understanding of each of the two systems, and also for filling some of the gaps left open and unresolved by legislative texts. It might also offer some contribution in view of a more general analysis of the key-issues that those who design the systems meant to govern the European economy should consider. These systems must come to terms with the need to combine and coordinate EU and national institutional actors that, by applying EU and national laws, act both at EU and national levels. As our analysis will touch upon the issues that originated the two frameworks at stake, bringing to their current institutional configuration, we will create a starting point for further research to draw upon commonalities and differences and determine what the best model is for any other area of law.

## Apples and oranges: what makes banking supervision and competition law enforcement inherently different?

Traditionally, and very much like apples and oranges, banking supervision and competition law, despite being both two areas of law governing economic fact are different for several crucial reasons, namely (i) the differences between the activities carried out within those frameworks—i.e., banking supervision vs competition law enforcement; (ii) the differences between the subjects to whom these activities are directed—i.e., banks vs any economic agent running economic activities; and (iii) the historical contexts where EU institutions decided to create the combinations of authorities analyzed herein.

It is through an analysis of these differences though that we can better remarks what still are the commonalities between the two systems.

### (i) Systematic (year after year) approach v. top-down (case after case) approach

To start with, banking supervision aims at ensuring the safety and soundness of the European banking system, while increasing financial integration and stability. To this end, banking supervision takes place within a set of high-level priorities that, year after year, represent the focus of supervisory activities—and, as a consequence, of the compliance activities of the supervised financial institutions—considering the main risks that the system may run. Thus, banking supervision is flexible and open to a certain degree of discretion, because, to some extent, policy reasons play a role in identifying the main challenges that banks may need to address over time, some sooner, other later. Furthermore, banking supervision occurs on a continuum, i.e., on a daily basis, through the articulation of routine measures within a general framework of ongoing relations and dialogue between the competent authorities and the market players.

On the other hand, with a growing number of exceptions to which we will return later,^9^ the activity of an antitrust authority applying competition law mostly resembles the activity of a judge, not only when it comes to the ex post enforcement of the law about anti-competitive agreements and the law about abuses of dominance, but also when authorities may ex ante decide to authorize mergers. Namely, the use of antitrust rules to ensure competition within the Single Market leads to punishing firms that violate the prohibitions set out by two specific provisions, i.e., Article 101 and Article 102 of the TFEU, and to guarantee that no anticompetitive merger within the meaning of Article 3 of Regulation 139/2004 will ever take place. Therefore, competition law enforcement is not very familiar with policy recommendations decided and re-discussed year after year, nor does it require the existence of an ongoing relationship between the antitrust authority at stake and the firms involved in the alleged violations. Quite independently from the identification of industries and sectors in need of competition, the enforcement of antitrust rules takes place case after case, because of a complaint, a self-complaint, an ex-officio investigation or, when it comes to mergers, because of a notification. Thus, when compared with the systematic and integrated nature of banking supervision, antitrust enforcement appears much more rhapsodic and top-down.

### (ii) Sector-specific (authorized subjects) v. across-industry (undertakings)

Coming to the second macroscopic difference distinguishing the frameworks analyzed herein, banking supervision concerns a given number of homogeneous subjects belonging to the same industry, i.e., the sole agents who, having obtained the authorization to carry out banking activities, belong to the banking industry. On the contrary, antitrust law applies to any “undertaking,” granted that any agent running an economic activity is an undertaking within the meaning of EU competition law.^10^ Thus, antitrust prohibitions and merger regulation relate to any sector, that is, to any natural persons and legal entities that, ranging from car dealers to pharmaceutical companies or bakery shops, operate at market conditions, even though they do not share the same features, interests, or risks. In short, whereas banking supervision may represent a tile in the more general banking regulation mosaic that, as such, answers to a kind of ex ante and pre-ordered design, competition enforcement is an across-industry tool, which is independent from any kind of sector-specific regulation and whose consistency is rooted in precedents that, over the years, have resulted in a sort of common European antitrust culture.

### (iii) Integrated framework v. filler framework

Finally, the mechanics with which banking supervision and competition law enforcements are carried out must be understood in light of the historical contexts within which they were conceived. As said above, in 2013 the creation of the SSM came together with the elaboration of harmonized principles and rules that were to shape the banking supervision that needed to be carried out in the Euro-zone. Thus, when it was conceived as an institutional mechanism of the new-born Banking Union, the SSM was as new as the function it was meant to carry out. On the contrary, the preexisting national banking supervision systems had very long histories in relation to both their institutional frameworks and the sets of principles and rules that they had to apply. In particular, even before the advent of the Single Rulebook, NCAs were already very familiar with EU banking law, because they already enforced EU Regulations applicable to credit institutions, as well as national legislation implementing EU Directives. Therefore, it is arguable that the current EU banking supervision system, which now pivots around the SSM, has not been called upon to fill a gap. Rather, it was *integrated* and *added* into the already existing national systems, mostly to reduce risks of regulatory arbitrage among Member States, and to face the difficulties related to the exercise of supervision over banks whose operations go far beyond national borders.^11^

On the other hand, the history of the enforcement of competition law is quite different and somehow less straightforward. Like the Single Rulebook, also the first EU Merger Regulation entered into force, in 1989, at a moment when Competition NCAs already applied their national rules on mergers (and only them, as before 1989 there were no EU provisions about merger control). However, the advent of the first EU Merger Regulation was not perceived as an act of integration or, in contrast, as an act of expropriation: rather, it was an “act of separation.” The first EU Merger Regulation, indeed, came together with a clear-cut division of roles between the EC and the Competition NCAs, which left Competition NCAs with their traditional areas of influence, while the EC advocated for itself the competence over the only cases capable of producing a major impact on the Single Market, that is, over the cases whose effects went far beyond national borders. Differently, at the very beginning of the European antitrust experience, if, on the one hand, the EC was already born and the European provisions about anti-competitive agreements and abuses of dominance were already in force, on the other hand, many Member States did not have either their own competition authorities or their own competition laws yet. This is true even though Regulation 17/62 (i.e., the first Regulation for the implementation of EU competition rules) had already envisaged a decentralized system for the application of such rules, by granting to the would-be Competition NCAs the power to enforce the first and second paragraphs of Article 101 and Article 102. In this sense, for many years, both EC and EU competition rules represented a twofold benchmark with which the Member States had to confront in order to create their own national antitrust authorities and rules. Also, even when certain Competition NCAs were finally put in place, they still did not enjoy the power to enforce the third paragraph of Article 101 because the EC feared that, absent a common antitrust culture, they could have bent this provision to the pursuit of their national interests. As a consequence, in 2003, the creation of the ECN was perceived as both an “act of full decentralization” and as an “act of separation”: the EC kept on playing its leading role in the enforcement of the provisions on anti-competitive agreements and abuses of dominance, whereas Competition NCAs were finally allowed to apply all the rules included in the Treaty’s provisions (that is, also the third paragraph of Article 101), thanks to a thick bulk of precedents and an established array of principles about the interpretation of Articles 101 and 102. In addition, Article 3 of Regulation 1/2003 clarified how EU and National competition rules should coexist: it establishes that, for cases subject to both EU and National laws, Member States are not precluded from adopting and applying *on their territory*—and only on their own territory—stricter national laws which prohibit or sanction agreements or unilateral conduct that are compatible with EU antitrust rules.

## The allocation of competences

The starting point of any analysis that wishes to compare the systems for banking supervision and the enforcement of competition law is their respective institutional frameworks. In particular, while focusing on the different solutions adopted to allocate competences, the analysis will also set out the choices made in relation to the applicable law and the explicit need to ensure a strict collaboration among the different layers of the same system of governance.^12^ In what follows, the paper will show how these context-related differences explain and justify some of the features that distinguish the systems of governance designed to administrate banking supervision and competition law enforcement. At the same time, the two systems show convergence in solving the issue of centralization vis-à-vis decentralization in EU economic governance matters: they, indeed, elaborate a solution where the balance is struck between centralizing and decentralizing, by way of an allocation of competences and cooperation mechanism between EU and National institutions and authorities. As the paper will try to show that, rather than re-thinking the substantial approach to be found in each of the systems—ex-post intervention for competition matters; managerial, regulatory regime, characterized by tight supervision for the SSM^13^—they offer a good and viable example of how to work out and solve the typical issue to be found in EU governance matters, that is the allocation of competences among Member States and EU institutions.

This chapter analyzes the cornerstone provisions of the frameworks and how they allocate competences between the different authorities that revolve around them.

Firstly, we will look at the dividing line between the ECB and the NCAs in the Single Supervisory Mechanism. Secondly, we will delve into EU merger law and the allocation of responsibilities between the EC and the NCA. We conclude with a similar analysis of the distribution of functions within the ECN.

### (i) The SSM

Concerning the SSM, the core provisions that we intend to focus upon for the purposes of these notes relate to the division of competences between the ECB and NCAs. Embedded in Council Regulation (EU) No 1024/2013 of 15 October 2013 (the SSM Regulation, or simply “SSMR”), such provisions operate in a twofold way: on the one hand, they set out the *general criteria* that mark the dividing line between the supervision carried out by the ECB, and the one that remains entrusted to NCAs; on the other hand, they trace the *allocation of competences* that, in concrete terms, results from such demarcation. This is the result of the compromise between the forces towards centralization—underpinning the entire Banking Union project and the SSM—and those towards decentralization, which were intensely confronted during the discussions that led to the final approval of the Regulation.

As to the dividing line between the ECB and NCAs, the choice that was made in the SSMR is, first of all (albeit not exclusively—see below) based on the *dimension* of the credit institution, whereby “dimension” is intended as a quantitative measure. The so-called significant banks, which exceed the thresholds established by the SSMR,^14^ are therefore subject to the ECB’s direct (and exclusive)^15^ supervision, save for those matters that the SSMR itself does not address at all, and leaves in the hands of NCAs^16^ (i.e., anti-money laundering and consumer protection^17^).

Banks that fall below the thresholds set out in the Regulation (the so-called less significant banks) are subject to a different regime, where there remains a role (which needs, however, to be clarified) for NCAs. In light of this distinction, the more practical allocation of competences between the ECB and NCAs is defined by Articles 4 and 6 of the SSMR, which, in part, follow a different approach.^18^ The ECB is, in fact, responsible for carrying out certain fundamental tasks with regard to *all* credit institutions that fall within the SSM (basically, all banks in the Euro area),^19^ irrespective of their sizes: such tasks are, in particular, the granting of the banking license and supervision over significant holdings in the capital of credit institutions. As anticipated, the reason for this articulation of competences is to be found in the need to reach a political agreement on the final text of the Regulation, that was intended to foster a full centralization of banking supervision in the Euro area, but resulted in a more acceptable compromise; however, it also lies in the fact that access to the market (whether directly, by way of a new license, or indirectly through the acquisition of qualified holdings) is a key point for supervision and therefore justifies a higher degree of centralization.

For matters referred to in Article 4 of the SSMR, other than those aforementioned, Article 6(6) of the SSMR allocates competences between the ECB and NCAs, following the distinction between significant and less significant banks: for significant banks, therefore, supervision is carried out by the ECB; for less significant banks, supervision is in principle allocated to NCAs.^20^

The relationship between the ECB and NCAs is, however, much more complex than what can be gathered from the simple distinction between significant and less significant credit institutions, as it depends on the complex network of cooperation and interchange between national and EU levels within the SSM. There are basically six different approaches and methods that can be gathered from the SSM in terms of relationship between the ECB and NCAs that show the great diversity of the scheme.^21^ Within the SSM: (i) the ECB directly applies supranational EU rules on prudential supervision, with the support provided by national authorities; (ii) the ECB, in the exercise of its tasks, instructs NCAs to exercise their respective supervisory powers, under national law, in accordance with national legislation; (iii) the ECB shares on a stable basis the exercise of its functions with national authorities, and, as a consequence, its administrative action is often structured according to mixed or composite procedures; (iv) in particular for less significant credit institutions, the ECB carries out its supervisory functions by using various tools to guide and intervene on actions taken by NCAs. Ultimately, the ECB also has the power to substitute NCAs; this gives way to a decentralized relationship, as stated by the Court of Justice of the European Union ("CJEU") in the *Landeskreditbank* case; (v) the ECB has a parallel and exclusive competence to the one exercised primarily by national authorities in a decentralized form; and (vi) finally, under an entirely original solution, the ECB directly applies national legislation that results from the implementation of EC Law (see Article 4, paragraph 3, SSMR). A full commentary and/or description of each of these different methods adopted within the SSM would imply a discourse far too long and complex for these pages: for our purpose, it is however useful to single them out, in order to highlight the complexities and nuances of the SSM.^22^ The allocation of competences between the ECB and NCAs is also not devoid of problematic aspects, as clearly shown in the landmark *Landeskreditbank* case and its implications (see below).

### (ii) Mergers

Some of the fundamental features of the SSM can also be found in the institutional framework governing the application of EU merger law and, in particular, in the rules and criteria according to which the EC and the competition NCAs divided their competences in the enforcement of merger control.

The legal basis for merger control in the EU is Council Regulation (EC) 139/2004, which replaced Regulation (EEC) 4064/89: it governs both substantial and procedural issues concerning concentrations.^23^ Since to dismantle entities resulting from mergers and acquisitions is a difficult and expensive task, both Regulations have established a system of prior notifications, subject to the so-called one-stop-shop principle.

According to the latter, when dealing with mergers having a cross-border impact, EU and national competition laws cannot overlap and the EC and Competition NCAs cannot share concurrent jurisdiction. In merger cases, it is therefore essential—and functional for the identification of the competent jurisdiction—to first identify the relevant, applicable law: whether EU or national. Namely, concentrations with a “Community dimension” fall within the sole scope of the EU competition law, and under the *exclusive* jurisdiction of the EC: they must be notified only to the EC; they are scrutinized according to the rules of the specific merger regulation in force, and they cannot be subject to any national Competition Law applied by Competition NCAs. Differently, concentrations that do not have a “Community dimension” may (or may not) be subject to national competition laws, which may (or may not) impose a prior notification to be addressed to a specific Competition NCA. Thus, as to merger control, there is a two-way relationship between the public authority in charge of the specific case at stake and the applied piece of law: the EC enforces only the EU law and each Competition NCAs applies only its own national competition law.

Pursuant to Articles 1(2) and 1(3), mergers have said “Community dimension” only if they involve companies reaching certain turnover thresholds.^24^ Therefore, over the years, the one-stop-shop principle has been hugely appreciated because, by preventing multiple merger control notifications and the consequent parallel actions, and by providing firms with fixed turnover-related criteria to identify the relevant jurisdiction, it has reduced costs, saved time, and increased legal certainty. To put it differently, the handling of a merger by a single competition authority—either the EC applying EU law or one Competition NCA applying its own national law—has enhanced administrative efficiency, avoided duplication and fragmentation of enforcement efforts, and eliminated the risk of conflicting decisions resulting from the concurrent assessment of the same transaction by a number of competition authorities following diverse legal regimes.

Admittedly, though, there may be exceptional cases where competition authorities act in derogation of this clear division of competences, even if each of them continues to apply its own competition law. For instance, as provided for in Articles 4(4) and 9 of Regulation 139/2004, it may happen that, prior to (or even after) the notification of a concentration with a EU dimension, the concentration is deemed (or found) to significantly affect competition in a national market which presents all the characteristics of a distinct market. In such a situation, it may happen that the EC refers the whole or part of the case to the competent national authority, which will then apply its national competition law. Likewise, as the famous and celebrated acquisition *Facebook-Whatsapp* shows,^25^ it may happen that one merger, which falls below the turnover thresholds referred to in Regulation 139/2010, may qualify for examination under a number of national merger control systems. As said, multiple notifications of the same transaction are costly and may result in conflicting decisions. Therefore, Article 4(5) of Regulation 139/2004 regulates the case of an undertaking that, prior to the notification, asks the EC to be in charge of the merger. If none of the interested competition NCAs disagrees, the EC will hence be the only authority authorized to receive the notification and, as a consequence, to apply EU law: for sure, in such a situation, the EC plays a leading and standardizing role, by deciding in one specific way a case that potentially, by affecting many national jurisdictions, could have given rise to conflicting assessments. Finally, there may be cases where one specific NCA believes the EC to be in the best position possible to carry out the investigation and thus refers the case to it, as described in Article 22 of Regulation 139/2004. Again, though the neat division of competences, there are cases where Competition NCAs do recognize the authority of the EC and, accordingly, its superior role.

In all referral cases, the EC and Competition NCAs retain a considerable margin of discretion in deciding whether to refer cases falling within their “original jurisdiction,” or whether to accept to deal with cases not falling within their “original jurisdiction.” In other words, nothing in Regulation 139/2004 prevents the EC from playing a guiding role, also because it must work in a close and constant liaison with Competition NCAs to form a network of public authorities (e.g., Article 19(2) of Regulation 139/2004). Each authority in the network applies its own respective laws, shares information and consults with other authorities, with the ultimate intent of ensuring not only that multiple notifications of a given concentration are avoided to the greatest extent possible (that is, that the one-stop-shop principle is respected), but also that the case at stake is dealt with by the most appropriate authority, as the principle of subsidiarity establishes.

In summary, when it comes to the European framework for the control of mergers, reference to the firms’ turnover is a clear and unquestionable quantitative parameter; this is similar to the approach taken by the SSM that, in identifying the “significance” of a credit institution looks, first of all, and as the most significant criteria, at a precise figure (in the latter case, not the turnover but the credit institution’s assets). These figures are thus assumed to be a good proxy of the effects that the phenomenon under scrutiny may produce and, in particular, of the impact and relevance that a certain concern may have on, or for, the EU market. There are, however, distinctions and specific points that need to be singled out. Within the SSM, the ECB can claim direct supervisory competence on the basis of a set of criteria—including following a notification by an NCA and a “comprehensive assessment” by the ECB; or on the grounds that an institution has asked for financial assistance; or if the ECB deems it necessary to “ensure consistent application of high supervisory standards” (Article 6(5)(b) SSMR). The ECB can also decide to reclassify an institution as “less significant” based on “particular circumstances” (Article 6(4) SSMR).

The distribution of competences between the ECB and NCAs is therefore complex and multi-variable. In competition law, there may be circumstances where, taking into consideration the specific characteristics of the case as well as the tools and expertise available, such a neat criterion is not sufficient to identify the most appropriate authority for dealing with the merger in question. Therefore, Regulation 139/2004 provides for the described referral mechanism that is meant to re-attribute cases by the Commission to Member States and vice versa.^26^

### (iii) The ECN

Articles 101 and 102 TFEU forbid anti-competitive agreements and abuses of dominance. They must be applied to conducts impairing the trade among Member States and may be enforced not only by national judges (private enforcement), but also by the EC and Competition NCAs (public enforcement). For what matters the most here, the separation of competences between the EC and Competition NCAs has changed over time because of the perils contained in the third paragraph of Article 101, which allows the survival of unlawful agreements by way of derogation.^27^ From 1962 to 2004, both the EC and Competition NCAs were allowed to apply Articles 101(1) and 102, but only the former was authorized to apply Article 101(3): the main reason for this was to prevent the risk that existing Competition NCAs—actually, only a few—might use EU competition law to support their national champions and, more generally, the interests of their own Member States. In other words, during those years, against an already decentralized application of Articles 101(1) and 102, the enforcement of Article 101(3) was centralized in the hands of the EC, which was perceived as the true guarantor of the Single Market. However, such a quite centralized system,^28^ combined with the enlargement of the European Union, cast a significant workload upon the EC that, as a consequence, did not have enough resources to go after cartels, other significant agreements, and abuses. In addition, over the years, antitrust culture gained momentum in the different Member States of the Union, and a bulk of cases, decided by the EC and confirmed by the CJEU, began to exert binding effects over the activities carried out by the NCAs. Consequently, the risk of a nationally oriented and centrifugal application of EU competition law by Competition NCAs was reduced. Therefore, with Regulation 1/2003, Competition NCAs acquired the power to apply Article 101(3) and, overall, the enforcement of Articles 101 and 102 was entrusted to both the EC and the other Competition NCAs, being all parts of the ECN. To put it differently, nowadays, the cases that may affect trade between Member States are subject to the fully-fledged scrutiny of either the EC or Competition NCAs, while national cases remain subject to the sole jurisdiction of NCAs. This is why, today, it is common to read that Competition NCAs and the EC form a network of public authorities, which act in the public interest and cooperate closely in order to protect competition on the basis of common discussion and cooperation.^29^

This fully decentralized system has, however, urged: (a) the need to define the criteria according to which EU cases—that is, cases capable of impairing interstate commerce—are managed by the EC in lieu of Competition NCAs or vice versa and (b) the need to ensure a uniform application of EU Law as so to guarantee its effect utile in the whole EU, irrespective of the specific acting authority.

Regarding the criteria,^30^ as it happens with mergers, the authority in charge should be the one “best placed” to guarantee the well-functioning of the market. But differently from what happens with mergers, no turnover criterion exists. Rather, an authority can be considered to be well placed to deal with a case if three *cumulative* conditions are met, i.e., “(i) the agreement or practice has substantial direct actual or foreseeable effects on competition within its territory, is implemented within or originates from its territory; (ii) the authority is able to effectively bring to an end the entire infringement, i.e., it can adopt a cease-and-desist order the effect of which will be sufficient to bring an end to the infringement and it can, where appropriate, sanction the infringement adequately; and (iii) the authority can gather, possibly with the assistance of other authorities, the evidence required to prove the infringement.”^31^ Therefore, parallel action by two or three competition NCAs may be appropriate where an agreement or practice has substantial effects on competition mainly in their respective territories and the action of only one NCA would not be sufficient to bring the entire infringement to an end and/or to sanction it adequately. In such a case, to coordinate their actions, the engaged Competition NCAs may find it useful to designate one of them as a lead authority and to delegate tasks to the lead authority such as for example the coordination of investigative measures, while each authority remains responsible for conducting its own proceedings.^32^ Finally, for the very same problems connected with parallel actions in merger cases, the EC will be presumptively considered well placed when a case affects three or more Member States *and* when the case at stake represents a “novelty” for the assessment of which the Commission’s authoritativeness would help to set the legal approach for the future,^33^ because of the leading role that the EC still plays within the ECN (see *infra*).

To comply with these allocation rules and ensure their efficient working, Articles 11(2) and 11(3) of Regulation 1/2003 lay down an obligation for all the authorities of the ECN: they must inform each other of their first formal investigative measure against one or more firms. In this way, indeed, the ECN may detect multiple procedures and address possible case re-allocation issues as soon as an authority starts investigating a case.

In addition, the well-working of the ECN—which has not only to do with the allocation of cases, but also with their handling—is rooted in an intense form of cooperation among all the authorities of the network.^34^ In particular, granted some safeguards,^35^ under Article 12 of Regulation 1/2003 such “cooperation” is achieved via a penetrating exchange of information, files, documents, evidence and opinions—these last about a great array of topics: from questions concerning investigative powers to important general/policy issues. Furthermore, such an exchange may happen at various stages of the procedures in progress: not only before cases are allocated; not only during the fact-finding phase of the cases; but also before infringement and commitment decisions are taken. Indeed, the ultimate goal of this flux of information, files, documents, evidence and opinions is meant to pursue a twofold goal: making available the best information possible to the authority actually in charge, as so to guarantee an effective enforcement of competition law, and ensuring a uniform interpretation and application of Articles 101 and 102, to avoid inconsistencies, that is, the case of the same practices subject to different approaches.^36^

## The leadership state of play: the ECB and the EC as "princes inter pares" within the SSM and the ECN

As seen above, the SSM and the ECN share similar concerns in terms of distribution and allocation of competences between national and EU level. They both end up by setting out a complex combination of cooperation, centralization and of dialectical confrontation between the two levels. After an analysis of the allocation of their functions from a strictly operational standpoint, we will now look at how these competences are performed through the interactions of powers between different authorities and frameworks.

The SSM is, in itself, the result of quite a delicate compromise between centralization and cooperation. On the one hand, it clearly centralizes supervision over credit institutions at the EU level; on the other, it sets out various rules that aim at ensuring, and enhancing, cooperation and mutual support between EU and national levels. It is indeed no coincidence that the SSM was immediately noted, after its introduction, for the strong accent that it places on cooperation between the ECB and NCAs.^37^ Within the SSM, the distinction between the EU and national level seems to be clearly drawn by that between significant and non-significant banks, save for the exceptions already recalled. There is, however, a possible derogation, based upon the existence of “particular circumstances” that might allow an otherwise significant bank not be treated as such, and therefore remain subject to supervision by its NCA, instead of the ECB.^38^ The issue related to the interpretation of the meaning of “particular circumstances” was intensely debated in the *Landeskreditbank* case, settled by the CJEU on 8 May 2019.^39^ By providing a narrow interpretation of that expression, the CJEU ended up also stating that, within the SSM, the ECB is entrusted with tasks and powers of prudential supervision over *all credit institutions falling within the scope of the SSM*. While, for significant banks, the ECB directly exercises tasks and powers, for less-significant ones, the effective exercise of supervisory powers, in any case vested upon the ECB, is “decentralized” to the NCAs. The conceptual framework arising from the decision in the *Landeskreditbank* case enhances, therefore, the central role of the ECB within the SSM, somewhat blurring the distinction between supervision over significant and less significant credit institutions, and the consequent division of competences between the ECB and the NCAs. At the same time, the relationship between the ECB and NCAs is articulated along multiple (at least six) strands of actions and interactions, as clarified above. The case is a striking example of how, on the one side, the issue of finding a proper balance between centralization and decentralization can be complex when building an effective system of EU/Member state governance, and of the essential clarifying role that may be played, in this context, by the EU’s Courts. Within the SSM, it is clear that, after the decision in Case C-450/17, centralization of the governance system of prudential supervision is a strong driving force that influences the interpretation of the relevant EU Legislative texts, reducing the space left to decentralization and National Authorities. However, this does not mean that decentralization is a moot concept in the context of the SSMR: rather, it needs to be framed and understood in the context of: (i) the allocation of competences between the ECB and NCA; (ii) the network of cooperation mechanisms and systems within the SSM, and (iii) the fact that, ultimately, the ECB is responsible for the entire, proper functioning of the system. A striking example, in other words, of allocation of competences among Member States and EU Institutions, aimed at tackling the complex issues of Banking prudential supervision.

As to the application of Articles 101 and 102, the supremacy of the EC within the ECN has never been questioned, probably because for more than 40 years the EC was exclusively entitled to enforce EU competition law in relation to the most difficult cases, that is, in relation to unlawful agreements to be authorized. With the advent of Regulation 1/2003, a system of parallel competences might have weakened the position of the EC. However, the system introduced by Regulation 1/2003 eventually produced an opposite result. Today, the EC is the *princeps inter pares* within the ECN: it retains its traditional leading role in the enforcement of EU competition rules,^40^ because not only does it intervene in particularly relevant cases,^41^ to set the “right” legal approach,^42^ also there are scenarios where the EC can advocate cases that fall within the jurisdiction(s) of Competition NCAs.^43^

In addition, not only does Regulation 1/2003 impose an intense exchange of information between the EC and Competition NCAs,^44^ also it provides the EC with a number of indirect tools that reinforce its privileged position within the ECN. In order to foster the efficient allocation of cases, Article 11 of Regulation 1/2003 imposes notification duties on Competition NCAs: when acting under Article 101 or Article 102 TFEU, they have to inform the EC by writing before, or without delay, after commencing the first formal investigative measure. According to Article 11(4) of Regulation 1/2003, no later than 30 days before the adoption of a commitment or infringement decision, competition NCAs must inform the EC, indicating the proposed course of action. This allows the EC to informally intervene in a proceeding before it is brought to an end, thus providing Competition NCAs different kinds of inputs, including the suggestion to re-examine the line taken with respect to particular allegations.^45^ Article 11(6) of Regulation 1/2003 entrusts the EC with formal powers to counter risks of misalignment within the system, giving it the possibility of initiating proceedings in the same case at any time during the investigation phase. The EC may also activate the same procedure and take over a case triggered by a national authority.^46^ In both cases, the intervention of the EC deprives the Competition NCA of its competence.^47^

Besides, irrespective of case allocation within the ECN, the Commission is directly responsible for guiding EU competition policy (see infra), by adopting communications aimed at providing priorities and general guidance for undertakings. To be sure, the Communications of the EC do not bound Competition NCAs, who enjoy a certain (and physiological) discretionary power in applying EU competition law. Nevertheless, so far the Communications published restate the law as it results not only from EC’s decisions, but—and more importantly—from the judgments of the CJEU. Therefore, as the Court is the final interpreter of the Treaty, the actions of Competition NCAs are consistent with the Communications, not because they are supposed to be their addressees, but because the Competition NCAs enforcing EU competition law cannot disregard the EU case law.

In conclusion, from the analysis above it seems that any multilayered system meant to govern the economy does require a higher ranking authority called to render the final word to more complex scenarios. The EC is the leader within the framework for the enforcement of competition law, whereas the ECB is the leader in governing the banking system.

## Different frameworks and different languages: "competences" and "powers" as the keywords of banking supervision and "uniformity" as the basis of antitrust enforcement

In light of the above, it is reasonable to wonder how scholars have conceptualized the legal frameworks governing the banking system and the enforcement of competition law.

That the SSM is an unprecedented model of integration between EU institutions and those of the Member States is a fact by now well established. The very name given to the SSM by its founding Regulation—a “mechanism”—falls outside traditional notions, so that the interpreter wonders whether the “mechanism” is, in fact, something different from the individual components that are part of it, or whether the term has a merely descriptive value.^48^

Looking at the prevailing literature on the SSM—at least before the *Landeskreditbank* decision—the positions expressed and the formulas used by interpreters to describe or, better, to frame the SSM, and the relevant division of competences between the ECB and NCAs, were quite diversified. Soon after the introduction of the SSM, there was a strong tendency to highlight the limitations to the ECB’s supervision over non-significant banks, and to place the SSM in a perspective where emphasis fell mainly on cooperation between supervisory authorities.^49^ Without claiming to completeness, among the various positions—which prove the richness of the current debate—the following can be found: (i) the position of those likely to be concerned, above all, with the need to highlight the profiles relating to cooperation and coordination within the SSM^50^; (ii) views describing the SSM in terms of “hub and spoke” schemes^51^; (iii) the positions of those considering that the ECB has a general “directive” function within the system^52^; (iv) the position of those using—not without difficulty—the more traditional categories of “direct” and “indirect” administration^53^; (v) the opinion of those attributing to the ECB, more simply, a role of general “oversight” with regard to the supervision carried out by the NCAs on non-significant banks^54^; (vi) the position of those distinguishing between “competences” and “powers”^55^; (vii) the reconstruction of the SSM through the use of categories proper to the models of administrative integration^56^; (viii) the position of those speaking of a “composite” system touching the three different profiles of regulations, administration, and judicial review^57^; and (ix) the position of those adopting a more descriptive approach, avoiding one more systematic.^58^

As to the enforcement of competition law, the conceptualization that revolves around the distinction between tasks and powers is not the one that best describes the relationships between the EC and the Competition NCAs. Arguably, it is easier to frame these relationships within the scheme of a decentralized application of Articles 101 and 102, which must be capable of guaranteeing the same useful effect beyond the subject who practically proceeds to pursue the anticompetitive behaviors at stake.^59^ In other words, assuming that the protection of competition in the Single Market can be conceptualized as the common “task”—or, better, as the common “goal”—of all antitrust authorities involved in the ECN, and considering that the “power” to apply Articles 101 and 102 follows the competence criteria mentioned above, the real challenge that the decentralized application of European antitrust law poses is to ensure that the actions of the Commission and NCAs are equally effective. Therefore, over the years, the main effort has been devoted to harmonize various procedural issues—such as the powers of investigations of the authorities, the rights of defense of the undertakings, the fines to be applied—as so to guarantee that antitrust proceedings are always the same, regardless of whether the anti-competitive practice is pursued by a Competition NCAs or the Commission.

Also, the enactment of Directive 2019/1—the so-called ECN + 60—can be explained in light of this need for uniformity. Indeed, this Directive sets out harmonization rules to ensure that Competition NCAS have the necessary guarantees of independence, resources, enforcement and fining powers to be able to effectively apply Articles 101 and 102 TFEU, so that competition in the internal market is not distorted and that consumers and undertakings are not put at a disadvantage by national laws and measures which prevent Competition NCAs from being effective enforcers. In other words, the ECN + aims to ensure that, when applying the same legal basis, i.e., EC antitrust rules, national competition authorities enjoy the very same appropriate enforcement tools to bring about a genuine common competition enforcement area. To that end, the proposal provides for minimum guarantees and standards to empower national competition authorities to reach their full potential.

That said, one can speculate in two directions. On the one hand, it can be observed that the uniformity required in the antitrust context might also be required in relation to banking supervision: just as an undertaking subject to Article 101 or 102 wants to be judged in the same way whether the judgment is made by a Competition NCA or by the EC, so a bank should want to be supervised in the same way whether the supervision is carried out by a NCA or by the ECB. On the other hand, on closer inspection, the archetype according to which “tasks” and “powers” are born united and are initially attributed to the same institutional actor, only to be subsequently separated (or even reunited) as the need arises, can also be repeated with reference to the antitrust context. In fact, we can state that the “tasks” and “powers” linked to the application of Articles 101 and 102 were, through Regulation 17/62 and Regulation 1/2003, conceived for the Commission and contextually decentralized to the Competition NCAs, partially in 1962 and totally in 2003.

## The taxonomy of the legal acts available within the SSM and ECN

Authorities can govern markets by using different legal tools that, in turn, pursue different goals and produce different effects.

At one antipode, there are EU Regulations. By looking at the cases of the SSM and the ECN, it is immediately clear that only the ECB and the EC—not by chance, “princeps inter pares”—may issue EU Regulations to set binding and uniform rules, directly and equally applicable in the entire European Union. For example, despite some initial dispute over the legitimacy of this exercise,^61^ the ECB has already exerted such regulatory powers, in order to fill some of the gaps left open by the CRD IV and the CRR. For instance, the ECB issued a Regulation to fix the threshold for assessing the materiality of past due credit obligations^62^ and a Regulation for reporting supervisory financial information,^63^ with the final intent of creating a common and mandatory metric to make the economic and financial situations of European banks comparable with each other. The ECB is also the competent authority that exercises options and discretions under the relevant CRD IV framework.

On the antitrust side, instead, the EC has never found any resistance in exercising regulatory powers. Indeed, over the years, it has enacted several Regulations: from procedural ones, such as the Commission Regulation 773/2004/EC that governs the EC’ proceedings for the application of Articles 101 and 102,^64^ to substantial ones meant to explain how to interpret and enforce Article 101 in relation to some kinds of agreements.^65^ In other words, beyond organizing its own procedures, the EC has used EU regulations to introduce safe harbors for some categories of agreements, in order to increase legal certainty and reduce enforcement costs.

On a closer inspection, however, EU Regulations are expensive and rigid legal acts, which require a broad and strong political consensus that is usually achieved over long periods of time which, in turn, often cannot be reconciled with market developments, moments of crisis or, more generally, do not allow the ECB and the EC to be sufficiently flexible and detailed. Therefore, at the other antipode of the spectrum of the legal acts available to the SSM and ECN, there is a significant amount of *soft law* that, more generally, is equally abundant in the contexts of EU banking law and EU competition law. The discussion on the regulatory or quasi-regulatory role of the ECB and of the EC, as well as of the respective national authorities, must indeed take into account the role of soft law.

There is a vivid and articulated debate on the role and nature of soft law in EU Law, and especially in the context of EU Financial legislation, lastly addressed by CJEU in the seminal FBF Case (Case 911/19, Judgment of the Court, Grand Chamber, 15 July 2021).^66^

The controversy leading to the Court’s decision of July 15 2021 revolves around the Guidelines on product oversight and governance for banking retail products, issued by the European Banking Authority ("EBA") in 2016 (hereinafter also the “2016 Guidelines”). Following the “comply or explain” mechanism that is proper to the ESAs’ Guidelines, the French ACPR informed, in September 2017, throughout a Notice, that it would comply with the EBA’s Guidelines. The French Banking Federation (FBF) challenged the Notice published in this respect by the ACPR before the French *Conseil d’Etat*, arguing that the Guidelines had been issued by the EBA exceeding its powers and competences. The Court ruled, in line with its own precedents, that the EBA Guidelines are non-legally binding measures and, therefore, cannot be subject to a direct action for annulment in front of the Court, but can, instead, be reviewed in the context of a preliminary ruling procedure. The Court also ruled that the 2016 Guidelines are valid, and that the EBA did not exceed its competences in publishing them, even though they do not have a direct and explicit legal basis in the Legislation, since they are in line with the aims and objectives that the EBA pursues in its mandate. By reaching these conclusions, the Court stretched what (if anything at all) still remains of the doctrine enshrined in Meroni and Romano, and sets the stage for a greater role of soft law in EU Financial legislation.^67^ Soft law in the context of the SSM deserves, however, its own attention. Within the SSM, one finds a specific eco-system of supervisory and quasi-regulatory tools that pose even more complex issues of qualifications (i.e., identification of their legal nature): instructions, guidelines, compliance letters, guides, reports, recommendations, etc.

### a. ECB Guidelines and instructions

within the SSM, the ECB has the power to issue guidelines and instructions addressed to NCAs. Neither of them are legal acts, since they are not included in the catalogue defined in Article 132(1) of the TFEU and Article 34.1 of the Statute of the ESCB: nonetheless, they are legal instruments and are binding on the NCAs to which they are addressed. This is a first, relevant difference with similar instruments issued, for instance, by the European Supervisory Agencies ("ESAs").

More specifically, ECB instructions can be case-specific or general. Case-specific instructions are addressed to a NCA and require the latter to take a particular course of action in a specific situation. For instance, a certain instruction might order the NCA to adopt a certain decision, or another act, by using the powers that the national authority has on the basis of its own national law. The ECB may also adopt instructions addressed to the NCA of a Member State in close cooperation within the meaning of Article 7(1) of the SSMR. These instructions have their legal basis in Article 9(1), third sub-paragraph, of the SSMR, as further specified in Article 22 of the SSMR.

On the other hand, instructions can be general, i.e., not relating to an individual case but rather to a certain topic or subject. Article 6(5)(a) of the SSMR empowers the ECB to adopt general instructions governing the supervision of less significant institutions by the national competent authorities. These general instructions set out the general framework and the main rules to be implemented by the national competent authority in relation to a certain topic or subject. This reflects the structure of the ECB, and the allocation of competences between the ECB and NCAs, as stated in the already cited *Landeskreditbank* decision of the CJEU, where the Court clarified that the ECB has the responsibility for, and oversight over, the entire system, in the context of which NCAs perform functions, inherently attributed to the ECB, on a decentralized basis.

The line between general instructions and guidelines might, in practice be uncertain: in theory the difference should lie in the fact that guidelines are of a quasi-regulatory nature, and therefore they are formulated in an abstract, and general way. General instructions, on the other hand, are addressed to one, or a subset of national competent authorities and in response to a specific supervisory topic: they apply to all less significant institutions subject to the supervision of NCAs, and which find themselves in the situation envisaged by the general instruction.

Based on the above, it is clear that there is also substantial difference between the guidelines, and general instructions of the ECB and Guidelines issued, for instance, by the European Banking Authority or by the other ESAs, which were recently discussed in the FBF Case by the CJEU (Case 911/19, Judgment of the Court, Grand Chamber, 15 July 2021).^68^

### b. Non-legally binding legal acts, instruments and documents

In addition to the legal acts and binding instruments described above, the ECB may also issue other non-binding instruments or documents. Instruments and policy documents published on the ECB’s website, while not imposing any obligations on third parties, may bind the ECB and therefore create legitimate expectations as to how European banking supervision will perform its supervisory tasks. To the extent it has created legitimate expectations, the ECB is bound to act accordingly, unless it provides justification: in this respect, the jurisprudence of the CJEU related to EU soft law, including the latest *FBF* Case would indeed be relevant to this category of acts, such as:

#### b.1. ECB Recommendations

ECB Recommendations are legal acts, devoid of binding effect. There are two types of ECB Recommendations:

- ECB Recommendations issued with the aim of stimulating certain legislative procedures at the Union level, leading to the enactment of complementary legislation; and
- ECB Recommendations used by the ECB to recommend that certain actions are taken by market participants. The ECB has made use of this type of recommendation on a number of occasions, including, lately, for dividend distribution policies.

The application of the Court’s jurisprudence to the ECB recommendations does not seem to raise any particular issues, as they should be subject to the same principles applicable to other recommendations issued by EU institutions.

#### b.2. Policy documents

Like most supervisors and central banks, the ECB publishes an array of other documents that are neither legal acts, nor legally binding. They include, for instance “Policy stances,” “Guidances,” “Joint Supervisory Standards,” “Methodologies,” “Guides” or “Letters.” Apart from the letters to Members of the European Parliament from either the President of the ECB or the Chair of the Supervisory Board, of which there have been several to date, these other documents can be divided into three groups: guides, reports, and letters to CEOs of credit institutions. Each of them deserves its own comments:

#### b.2.1. Guides

Their aim is to ensure consistency between, and equal treatment of, significant credit institutions, by setting out the details of processes applied by the ECB in the exercise of its supervisory tasks. Examples of these guides include the *Guide to assessments of licence applications*, the *Guide to internal models*, the *Guides to the internal liquidity adequacy assessment process (ILAAP) and the internal capital adequacy assessment process (ICAAP)*, the *Guide to on-site inspections and internal model investigations* and the *Guide to fit and proper assessments*. These Guides are also regularly updated. The scope of the Guides, which are periodically updated, may be (depending on the circumstances): (i) to clarify how the ECB intends to apply relevant Union law; (ii) to describe the details of processes applied by the ECB in carrying out its supervisory tasks; or (iii) to describe how the ECB would expect a credit institution to act in complying with EU law. Guides or guidance cannot and should not aim to create new obligations or requirements on credit institutions. In principle, they should therefore fit snugly into the conclusions of the FBF decision, unless their wording and their analysis, on an objective plan, shows differently;

#### b.2.2. Reports

The ECB, as any other supervisor, publishes reports with the aim to inform the general public of supervisory activities it performs. These reports are usually produced only once, save for the ECB Annual Report on supervisory activities. These documents are unlikely to be considered as legally binding or as able to produce binding effects, even though they are highly respected and considered as very valuable sources of information;

#### b.2.3. Letters

The ECB publishes letters that it sends out to CEOs of credit institutions. Typically, they are addressed to all credit institutions and contain general, non-confidential information relevant for all credit institutions or a large portion of them. Only non-confidential letters to credit institutions are made public, and usually only those either addressed to or relevant for all credit institutions may be considered for publication, but there may be exceptions. Letters to CEOs may also merely contain information on upcoming publications or supervisory exercises.

Although most of the documents made public by the ECB in the exercise of its supervisory tasks can be placed in one of the broad categories listed above, not all of such documents fit perfectly into one of these categories, and some may have a hybrid nature. A report, for instance, may effectively formulate supervisory expectations (see, for instance, the SSM thematic review on profitability and business models of September 2018, formally entitled as a mere “report”). This makes the picture complex, and difficult at times to properly classify.

As to the pieces of soft law that dwell in the realm of EU competition law, over the years, the EC has enacted several Notices and Guidelines where, on the one hand, the EC has restated and systematized not only its decisions, but also the case law of the CJEU and, on the other hand, it has illustrated the principles and main ideas that lie behind its conceptualization of competition law. In particular, in addition to notices about procedures and fines,^69^ the EC has elaborated several communications on substantive matters, such as: the notion of the relevant market,^70^ the *de minimis* effect,^71^ the criteria for the application of Article 101(3),^72^ the notion of interstate commerce^73^; or the notion of control.^74^ Authorities, judges, and the whole antitrust community pay a lot of attention to these pieces of soft law for two reasons at least: first, because they contain the main principles that the CJEU has established in interpreting EU competition law; second, because they describe what the parties can expect from the EC. Indeed, as the CJEU has recently affirmed,^75^ the Communications of the EC set the boundaries of the Commission’s powers. Therefore, also the pieces of antitrust soft law play a significant role in ensuring the smooth working of the ECN, because they guarantee uniformity and reduce the degree of discretion possible in interpreting EU competition law.

More recently the EC and Competition NCAs have been issuing different kinds of guidelines and policy documents, specifically meant to set and explain their enforcing activities. The *Guidance about the Enforcement Priorities in Applying Article 102* of 2009 was the first act of this type^76^; lately, because of the exceptional circumstances triggered by the Coronavirus, there have been developed guidelines explaining how competition authorities can help firms deal with the crisis, by cooperating with each other in order to overcome the pandemic to the ultimate benefit of consumers.^77^ The EC is also willing to provide firms with ad hoc *comfort letters*,^78^ to guide single companies, associations and their legal advisors in relation to specific EU-wide cooperation initiatives that they may swiftly undertake to tackle the coronavirus pandemic, although their compatibility with EU competition rules may seem uncertain. All these acts seem to support the evidence that recently also competition authorities have started playing a regulatory role similar to that of banking supervisory authorities. Nevertheless, a clarification must be made. Measures adopted to manage the pandemic were clearly presented as temporary and exceptional, and therefore the EC and Competition NCAs did not found any difficulty in taking distance from the “more ordinary” enforcing priorities set forth in the 2009 Guidance, when they were called to adjudicate cases that did not fall among those priorities.^79^ Ultimately, given what was said above in relation to the ECB and national banking authorities that do perform regulatory activities, the time does not seem ripe to consider EU and National Competition authorities as fully-fledged regulatory bodies.

Finally, all the authorities engaged in the SSM and ECN may take *decisions*. In this regard, at least two issues deserve to be explored: the first regards the identification of the courts—EU vs national—with competence over the review of those decisions; the second concerns the kind of decisions that the authorities within the networks can take.

### (i) Who is accountable for the decisions taken and who can review them?

The institutions and authorities involved in the SSM and in the ECN collaborate, each in their own field, with the decision-making process: for example, each of them may investigate on behalf of the others^80^; as seen above, they may exchange information, files, documents, evidence, and opinions; furthermore, national authorities may (or even must) act on the basis of instructions set forth by the EU institutions. Such a collaboration may generate an issue of accountability: the addressees of the decisions resulting from this form of cooperation may wonder about who is actually responsible for the decisions taken and who, as a consequence, could be allowed to review them.^81^ In this regard, the experiences learned so far from the ECN and the SSM suggest distinguishing between two scenarios.

The first one is common to both banking supervision and the enforcement of EU competition law and regards the case where national authorities carry out acts that are preparatory to the ultimate decisions taken by the European institutions. In such a case, preparatory acts do not produce any autonomous effect on the parties.^82^ Thus, even if, theoretically, national courts would be competent for reviewing these preparatory acts,^83^ the parties could appeal only against the ultimate decisions, as these last are the only acts producing binding external effects on them. Even recently, the CJEU reiterated that when the ECB exercises an exclusive competence, no judicial review is available at national level over preparatory acts taken by the NCA. Therefore, if ultimate decisions were taken by the ECB, only EU courts are allowed to review them.^84^ On the other hand, nothing prevents European courts from finding that substantial legal flaws in a national preparatory act have become substantial legal flaws of the final European act.

The second scenario deserving consideration is, instead, specific for banking supervision and regards the case where it is not possible to distinguish between preparatory acts taken by national authorities and ultimate decisions taken by EU institutions, being only a single (definitive) act adopted by national authorities, albeit influenced by the ECB's stance. In the *Krohn* judgment (1987),^85^ the CJEU established the principle of liability of European institutions in the sole case where national authorities take decisions that not only mirror the principles and indications coming from EU institutions but result from their mandatory instructions. In other words, when NCAs do not enjoy any degree of discretion in carrying out their activities, the EU institutions remain accountable for the decisions taken by national authorities. By applying this principle within the SSM, if a party is affected by a national act adopted by an NCA in accordance with the ECB's mandatory instructions, the party should be entitled to seek judicial protection before the CJEU.^86^

### (ii) Every issue has its own decision

In general, authorities may steer firms to minimize harmful conduct; or they may intervene to educate and deter, so as to prevent future unlawful behaviors, or to help market operators finding a solution, to quickly cope with emerging problems.

Against this backdrop, one would expect a sort of opposition between the SSM and the ECN: whereas the regulatory rationale behind the former should result in supervisory decisions meant to guide banks on the basis of their daily activity, the adjudicatory rationale that lies at the core of the latter should justify infringement decisions meant to punish firms that have undertaken anticompetitive conduct. However, the array of decisions available within the two networks is much less simplistic than what this opposition suggests.

First, within both networks case-handlers take pure administrative *measures*, which serve the function of ensuring the proper working of the procedures in place. These measures are not decisions submitted to a formal adoption procedure, but technical acts of the many services and departments internal to the authorities involved in a specific case. For example, within the ECN, these measures consist in simple requests for information; simple inspection; access to file authorizations; extension of time-limits; transmission of documents; transmissions of information. Likewise, within the SSM, the ordinary functioning of prudential supervision implies similar measures.

Second, as it happens within the ECN, also in the context of the SSM one can identify infringement decisions with a clear deterrent character^87^ and, in particular, infringement decisions that come together with fines whose amount may even be so afflictive that the related decisions acquire a certain *coloration pénale*.^88^ Thus, as for the infringement decisions of the ECN, also the infringement decisions of the SSM must be taken in accordance with some fundamental principles and defensive rights, such as the principle of culpability; the principle of *ne bis in idem*; the right to remain silent; the principle of full jurisdiction; and the principle of the separation between investigative and decision-making functions. In addition, differently from supervisory or other regulatory decisions but equally to the antitrust infringement decisions, the infringement decisions imposing fines taken by the SSM must be subject to full judicial review, pursuant to Article 6 of the ECHR and Article 47 of the EU Charter of Fundamental Rights.

Third, as it happens within the SSM, also the authorities within the ECN may adopt acts meant to steer and guide firms and market actors. In addition to the panoply of instruments of soft law mentioned above, which clearly help firms to distinguish procompetitive from anticompetitive behaviors and sometimes set priorities and exceptional measures, since the adoption of Regulation 1/2003, the Competition NCAs and the EC may issue “commitment decisions.” The latter provide the antitrust authority with the opportunity of restoring effective competition on the market, without any need to conclude on the existence of an infringement or impose fines. Via commitment decisions, the antitrust authorities of the ECN voice their competition concerns, while the parties can come forward with commitments to address these concerns and, hence, solve the competitive issues under scrutiny without getting any declaration as to the unlawfulness of their behaviors and any fine.^89^

## Concluding remarks

The purpose of this paper was to compare the frameworks currently governing the functioning of the banking system and the enforcement of competition law in the European Union. More specifically, this paper sought to highlight: (i) what issues the designers of these systems had to face; (ii) what solutions they brought forward; and (iii) what caused the two different outcomes at stake.

Thus, to provide the Union with effective systems of economic governance, three main issues must be addressed.

The experience gained in the field of competition law suggests that those who design economic governance systems should, *first of all*, identify the law that is best placed to solve the problems at stake. For example, the application of EU competition law (in lieu of national competition law or together with it) depends on parameters and criteria that are meant to grasp the potential and actual “impact” of the case under scrutiny, so as to drive under EU law the cases of major importance. Indeed, Articles 101 and 102 apply when the conduct in question may undermine the commerce between Member States, while the EU Merger Regulation applies when the concentration under study has a “Community dimension.” Likewise, harmonized banking rules that are part of the EU Single Rulebook (in lieu of now-abrogated national banking rules or together with still-enforceable national banking rules) are meant to avoid regulatory arbitrage among Member States (as well as unfair competition among their national banks) in relation to key-issues of systemic importance, such as banks’ capital requirements or banks’ recovery and resolution procedures. Within the SSM, the ECB applies EU law, but also national law resulting from the implementation of EU law. National authorities, instead, apply EU law when it is directly applicable, and national law, regardless of whether the latter is the result or not of the implementation of EU law. In any case, national law must be applied consistently with EU law. This system should ensure high level of consistency and uniformity throughout the Union: in the Single Rulebook, both the ECB and NCAs should, in principle, find a set of common rules to apply consistently, both at the EU and national levels, addressing issues that, because of their impact on the EU market for banking products and services, are crucial in creating an effective Banking Union. However, the level of harmonization achieved by the Single Rulebook needs to be improved, since, in several areas, the margins of discretion and optionality left to Member States are too wide.

*Second*, any multilayer scheme apt to govern the economy of the EU should identify the competent authorities, that is, the authorities asked to intervene in order to serve the EU (and national) interests in the best way possible. The experiences analyzed above show that the following alternatives are possible: (*i*) akin to merger control, one may have a clear-cut distinction, under which EU institutions apply only EU law and national authorities apply only their national legislation; or (*ii*) akin to the ECN, one may have a decentralized application of EU law, where the latter is applied by both European institutions and national authorities; or, finally, (*iii*) like with the SSM, one may have a system which results from an “act of integration” in which one may even have the very peculiar case of an EU institution allowed to apply also national laws, as long as they are the result of the implementation of EU Law.

*Third*, as seen in both systems herein analyzed, to make the interplay between the EU and the national levels work, it is necessary to design *both* mechanisms of intense cooperation among the many agencies involved, *and* criteria for dispute resolution in cases of possible conflicts. For example, in order to ensure an efficient allocation and effective administration of cases, the authorities involved must communicate and exchange information, so as to provide the acting authority with the most exhaustive information possible and to identify the authority best placed to face the case. In particular, this last result can be achieved not only by fully exploiting the degree of flexibility included within the criteria meant to allocate competences, but also by recognizing a leading role to one specific institution to solve cases of conflicts, where they are possible. To be sure, the one-stop-shop principle underpinning the system meant to govern merger control has excluded from the outset any case of conflict, which on the contrary remain—at least, theoretically—still possible where the jurisdictions are concurrent, as it happens within the SSM and the ECN.

In more general terms, the comparison here developed shows that these governance systems identify a criterion that clearly triggers the operation at the centralized level, consisting of (broadly) quantitative measures (albeit other criteria are also employed), that ultimately single out a relevant “threshold” below which a certain topic or issue shifts into the hands of National institutions. Looking at the SSM and competition law, it is evident that the threshold criteria is important, but also non-conclusive, and needs to be integrated and adapted by using additional criteria. This approach (which ultimately turns to be multivariable) might be adopted in other sectors as well, as it provides, on the one side, clarity on the allocation between EU and national level, but also sufficient flexibility in order to properly address the specificities of different areas and topics. The initial discussions on the reform of the ESAs in 2017 somewhat mirrored the same rationale, as legislators intended to allocate competences between national and European authorities following a quantitative threshold mechanism, quite similar to the one in place for the SSM and the ECN (e.g., by providing the European Securities and Markets Authority ("ESMA") with new supervisory powers in relation to certain transactions or market players having an effective “EU relevance”). However, these initial ideas were not reflected in the reforms actually adopted in 2019.

Furthermore, the comparison shows that the preferred structure for the governance of relevant economic topics in the EU might indeed move in a direction which results in a combination of centralization and decentralization, organized around a threshold of direct EU relevance, with institutions and authorities operating at both levels in a coordinated way. This is, probably, one the most striking but also intellectually useful conclusions to be drawn from comparing the two apparently different mechanisms of economic governance: the acknowledgment of the fact that, ultimately, there can be a general infrastructure that is still capable of leaving sufficient space for adaptation and flexibility to address any relevant particularities stemming from the market.

The comparison also shows that EU competition enforcement and EU prudential supervision serve different goals, and also respond to different approaches in terms of policy: competition law is closer to a system of adjudication, and operates on an intermittent, case-by-case basis. Financial supervision is, instead, much more flexible and articulated in pursuing its goals and is performed on a continuous, ongoing basis. This difference in approach is ultimately logical, as it reflects the inherently different nature of the two policies. However, there are signs of osmosis in this respect: the need to ensure strict adherence to the rule of law in the context of EU banking law, together with the increasing attention to issues of accountability of EU supervisors, and the already significant cases brought to the attention of EU courts in those contexts, inoculates, within the SSM, elements that might in part fledge it to incorporate at least some core features typical of adjudication. On the other side, the antitrust framework is seeing the inoculation of elements (such as commitment decisions and guidelines setting priorities) that blend its traditionally adjudicatory nature, providing a more articulated texture than its traditional one.

In light of the above, the comparison between competition control and banking prudential supervision provides useful insights on what might be a general standard for EU economic governance in this respect: again, the point of equilibrium seems to result from a balancing of centralization and decentralization of structures. An ideal EU institutional architecture seems to require a two-level apparatus which is neither fully centralized, nor entirely decentralized, but rather articulates and blends both elements in reciprocity, so that national and European levels are constantly interconnected by cooperation arrangements. The formula for the distribution of prerogatives is not exact but should generally combine quantitative and qualitative measures to allocate powers and responsibilities amongst the relevant stakeholders.

Further research and observations need to be carried out in the future in order to develop a more comprehensive interdisciplinary analysis of the EU competition and banking supervision governance systems. Both systems are somehow “living organisms” that evolve as a consequence of different forces: evolution of administrative standards, judicial review and the role of the courts, changes in market structures and players, etc. To see how they move, and change, is a unique, albeit challenging, intellectual experience. We strongly believe that the dialogue between scholars and institutions belonging to the two fields should continue. The consolidation of the EU as a truly global system of economic governance should be carried out in full awareness of what has been achieved across different sectors and areas, and by identifying common or recurring patterns. It is these patterns that might provide the basis for further developments, as the Union's scope of action gradually extends to further areas.

## Footnotes

1 The SSM, at the moment of its introduction, was limited to the Euro area, due to the connection that exists between monetary policy and supervision over the financial system. However, the SSM is open to the accession of non-Euro countries, which can establish a system of close cooperation with the SSM according to the procedure set out by Article 7 of the Regulation. Bulgaria and Croatia joined European banking supervision through close cooperation in October 2020. Considering the link between supervision and monetary policy, economic theory follows the view that, when the Central Bank is the monetary actor, its involvement in supervision should be calibrated with prudence, so as to avoid hampering the effectiveness of monetary policy: the Central Bank’s involvement in supervision could become a vehicle that increases the risk of capture that the overall architecture of Central Bank independence should avoid. A cautious approach could however be followed, since theory doesn’t have clear-cut prescription on the optimal level of the Central Bank’s involvement in supervision. The most relevant contributions of the huge and growing literature on such topic provide contrasting recommendations: see, about supervisory tasks of Central Banks, Conti-Brown, P. and Lastra, M.R. (2018) Research Handbook on Central Banking, Edward Elgar.

2 The Single Rulebook is the backbone of the Banking Union regulation in the EU. It consists of legal acts with which all financial institutions in the EU must comply. Among other things, the Single Rulebook lays down capital requirements for credit institutions, ensures better protection for depositors, and regulates the prevention and management of bank failures. Its most relevant legal acts are Directive 2013/36/EU of 26 June 2013 on access to the activity of credit institutions and the prudential supervision of credit institutions and investment firms (CRD IV) and Regulation (EU) No 575/2013 of 26 June 2013 on prudential requirements for credit institutions and investment firms (CRR); the amended Directive 2014/49/EU of 16 April 2014 on deposit guarantee schemes (DGSD); and Directive 2014/59/EU of 15 May 2014 establishing a framework for the recovery and resolution of credit institutions and investment firms (BRRD).

3 See Cassese, S. (2014) La nuova architettura finanziaria europea, Dal Testo unico bancario all’Unione bancaria: tecniche normative e allocazione di poteri. In: Quaderni di ricerca giuridica della consulenza legale della Banca d’Italia, No. 75, 15:23. Most recently, on the architecture of the Banking Union, Zilioli, C. and Wojcik, K.-P. (2021), European Banking Union: a giant step towards European integration and a challenge for judicial review. In: Zilioli, C. and Wojcik, K.-P. (eds.) Judicial Review in the European Banking Union, Edward Elgar, 1:17.

4 EU law does not provide for a definition of NCAs. Nonetheless, it is possible to infer from Article 35 of Reg. 1/2003 that in the context of ECN an NCA is the body, or bodies, responsible for the effective application of competition law, regardless of their administrative or judicial character according their national legislation. In this article, they are referred to as “Competition NCAs”, in order to distinguish them from the NCAs under the SSMR.

5 Council Regulation (EC) No 139/2004 of 20 January 2004 on the control of concentrations between undertakings (the EC Merger Regulation), OJ L 24, 29.1.2004, 1–22.

6 Council Regulation (EC) No 1/2003 of 16 December 2002 on the implementation of the rules on competition laid down in Articles 81 and 82 of the Treaty, OJ L 1, 4.1.2003, 1. See, also, Commission Regulation (EC) No 773/2004 of 7 April 2004 relating to the conduct of proceedings by the Commission pursuant to Articles 81 and 82 of the EC Treaty, OJ L 123, 27.4.2004, 18, as amended by Commission Regulation (EC) No 622/2008 of 30 June 2008 amending Regulation (EC) No 773/2004, as regards the conduct of settlement procedures in cartel cases, OJ L 171, 1.7.2008, 3.

7 https://ec.europa.eu/competition/antitrust/nca.html.

8 A first glimpse at some of the issues discussed in this paper may be found in Pizzolla, A. (2018) The Role of the European Central Bank in the Single Supervisory Mechanism: A New Paradigm for EU Governance, E.L. Rev., 3-23.

9 See paragraph 5.

10 The notion of undertaking in EU competition law is traditionally described as functional, because its boundaries are defined in light of the goal that the EU institutions pursue when they apply Article 101 and 102 TFEU. In more detail, on the basis of the existing case law it can be argued that, to qualify a person as an undertaking, one must consider three issues. *First*, one must assess whether, in the specific scenario, it would be convenient for a natural or legal person to carry out an economic activity, i.e., an activity that consists of offering goods or services on a given market. See, e.g., C-41/90, *Klaus Höfner and Fritz Elser v. Macrotron GmbH*, ECLI:EU:C:1991:161, para 21; C-159/91 and C-160/91, *Poucet —Pistre*, ECLI:EU:C:1993:63, para 17; C-244/94, *Fédération française des sociétés d'assurances and others*, ECLI:EU:C:1995:392, para 14. *Second*, in qualifying a person as an undertaking, it is necessary to remember that for EU competition law the concept of undertaking is a relative one: the same person carrying out different activities may and may not, at the same time, be an undertaking, depending on the specific activity taken into consideration. See C-82/01 P, *Aéroports de Paris v. Commission*, ECLI:EU:C:2002:617, para 74 and C-49/07, *MOTOE*, ECLI:EU:C:2008:376, para 25. Finally, in characterizing a person as an undertaking a “criterion of the minimum efficient unit” must be respected. More explicitly, when faced with scenarios in which several persons are involved in a given practice, one must identify the minimum combination of natural and legal persons who are autonomously and independently engaged in that conduct. See, e.g., Odudu, O. and Bailey, D., (2014) The Single Economic Entity Doctrine in EU Competition Law, *Common Market Law Review*, 51:1721-1758 and Jones, A. (2012) The Boundaries of an Undertaking in EU Competition Law, *European Competition Journal*, 8: 301-331. Indeed, the most recent case law states that the notion of undertaking within the meaning of Article 101 TFEU refers to a single economic unit which consists of “a unitary organization of personal, material and immaterial elements which pursues on a stable basis a certain end of an economic nature [and] which may contribute to the realization of an infringement envisaged by that provision” —T-112/05, *Akzo Nobel NV vs Commission*, ECLI:EU:T:2007:381, paras 57-58; T-9/99, *HFB and others v. Commission*, ECLI:EU:T:2002:70, para 54; and T-11/89, *Shell v. Commission*, ECLI:EU:T:1992:33, para 311.

11 Arguably, the former Committee of Banking Supervisors (CEBS), established by the EC in 2004 through Decision 2004/5/EC and extinct in 2011, may be seen as a precursor of supervisory cooperation in the EU, including for exchange of information, even though it did not have the formal prerogatives and powers invested upon current-day institutions.

12 On the similarities between the two systems, see H. Schweitzer, Banking Union, cit., where the Author points out that “Given the EU’s experience in EU competition law enforcement, it does not come as a surprise that the ECB’s enforcement powers as foreseen in the SSM follow, in important respects, the model of Regulation 1/2003 on the implementation of Articles 101 and 102 TFEU. But the legitimacy requirements for strong enforcement powers within a strongly managerial model differ from the legitimacy requirements of enforcement powers in a rule-based model”, p. 230. On the basis of this remark, the Author proposes to substitute the current system of rule-based ex-ante and discretionary supervision on Banks, with a different approach based on high capital requirements (re-elaborating previous, similar proposals). The interesting proposal, however, does not seem to be, at the moment, in the radar of the most recent legislative initiatives of the UE in the banking supervisory field.

13 This is the suggestion set out, for instance, by H. Schweitzer in Banking Union and the European Economic Constitution: A Brief Comparison of Regulatory Styles in Banking Regulation and Competition Law, in The European. Banking Union and Constitution. Beacon for Advanced Integration or Death-Knell for Democracy?, edited by Stefan Grundmann and Hans-W. Micklitz. (Oxford: Hart Publishing, 2021, 214 ss.

14 According to Article 6(4) SSMR, the quantitative criterion, identified at the 30 billions of Euro, is not the only one. An entity is, in fact, considered significant also when one of the following other conditions is satisfied: (a) the ratio of its total assets over the GDP of the participating Member State of establishment exceeds 20%, unless the total value of its assets is below EUR 5 billion; (b) following a notification by its national competent authority that it considers such an institution of significant relevance with regard to the domestic economy, the ECB takes a decision confirming such significance following a comprehensive assessment by the ECB, including a balance-sheet assessment, of that credit institution. Furthermore: (c) the ECB may also, on its own initiative, consider an institution to be of significant relevance where it has established banking subsidiaries in more than one participating Member States and its cross-border assets or liabilities represent a significant part of its total assets or liabilities subject to the conditions laid down in the methodology; (d) those for which public financial assistance has been requested or received directly from the EFSF or the ESM shall not be considered less significant; (e) notwithstanding the previous subparagraphs, the ECB shall carry out the tasks conferred on it by this Regulation in respect of the three most significant credit institutions in each of the participating Member States, unless justified by particular circumstances; (f) when necessary to ensure consistent application of high supervisory standards, the ECB may at any time, on its own initiative after consulting with national competent authorities or upon request by a national competent authority, decide to exercise directly itself all the relevant powers for one or more credit institutions referred to in paragraph 4, including in the case where financial assistance has been requested or received indirectly from the EFSF or the ESM. For a wide assessment, see Gasparri, G. (2017) I nuovi assetti istituzionali della vigilanza europea sul mercato finanziario e sul sistema bancario. Quadro di sintesi e problemi aperti, Quaderni giuridici Consob.

15 The ECB's direct supervision encompasses the action of the so-called joint supervisory teams ("JSTs"), an ad hoc team formed by ECB and NCA staff supervisors. A JST is established for each significant credit institution under the SSM and is responsible for carrying out its ongoing prudential supervision. Typically, a JST is composed of a coordinator at the ECB, national sub-coordinators and a team of experts, whose size varies in proportion to the nature, size and complexity of the institution. For this reason, even though there is an amount of cooperation between the ECB and NCAs (see footnote 35), the JST structure is still part of the SSM's internal organization under the ECB and the high-level decisions taken by JSTs are all dependent on the ultimate approval from the Governing Council and Supervisory Board.

16 The perimeter is established by Recital n. 28 of the SSMR, which reads as follows: “Supervisory tasks not conferred on the ECB should remain with the national authorities. Those tasks should include the power to receive notifications from credit institutions in relation to the right of establishment and the free provision of services, to supervise bodies which are not covered by the definition of credit institutions under Union law but which are supervised as credit institutions under national law, to supervise credit institutions from third countries establishing a branch or providing cross-border services in the Union, to supervise payments services, to carry out day-to-day verifications of credit institutions, to carry out the function of competent authorities over credit institutions in relation to markets in financial instruments, the prevention of the use of the financial system for the purpose of money laundering and terrorist financing and consumer protection”.

17 The reason for this carve-out from the general competence of the SSMR is the by-product of the political debate that preceded the adoption of the Regulation, but also justifiable in terms of market structure and of the fact that those topics are closely linked to local market structures.

18 An analytical picture of the allocation of competences between the ECB and National Authorities is offered by D’Ambrosio, R. (2013) Due process and safeguards of persons subject to SSM supervisory and sanctioning proceedings, Quaderni di ricerca giuridica, Banca d’Italia, Rome.

19 In addition to Bulgaria and Croatia, as per footnote 1.

20 The peculiar structure of the SSM is well reflected in the controversy, raised in front of the German Constitutional Court, relating to the compatibility of the SSM – together with other texts of EU Secondary Law on the Banking Union – with the Treaty and the Constitutional counter-limits of Member States. The matter concerns, among others, the exercise of the supervisory functions attributed to the ECB by Regulation 1024/2013 establishing the SSM. The case developed in parallel in front of the CJEU) and in front of the Bundesverfassungsgericht (“BVerfG”): see Case C-450/17 P, *Landeskreditbank Baden-Württemberg—Förderbank v European Central Bank*, ECLI:EU:C:2019:372; and BVerfG, 30 July 2019, BvR 1685/14, 2 BvR 2631/14. The point of greatest contact between the judgment of the BVerfG in the Banking Union Case and that of the CJEU in the Landeskreditbank case concerns precisely the relationship between Article 4 and Article. 6 of the SSMR, i.e. the division of competences between the ECB and the NCAs. Extensively on the relation between the two judgments, F. Annunziata, M. Lamandini, and D. R. Muñoz, Weiss and EU Union Banking Law. A Test for the Fundamental Principles of the Treaty (July 13,2020). European Banking Institute Working Paper Series 2020—no. 67, available at SSRN: https://ssrn.com/abstract=3650430 or http://dx.doi.org/10.2139/ssrn.3650430.

21 See Magliari, A. (2020) Vigilanza bancaria e integrazione europea, ESI, p. 183.

22 For a comprehensive overview, see Magliari, A. (2020) Vigilanza bancaria e integrazione europea, ESI, p. 183, and the abundant literature cited therein.

23 Pursuant to Art. 3(1) of Reg. 139/2010, “A concentration shall be deemed to arise where a change of control on a lasting basis results from: (a) the merger of two or more previously independent undertakings or parts of undertakings, or (b) the acquisition, by one or more persons already controlling at least one undertaking, or by one or more undertakings, whether by purchase of securities or assets, by contract or by any other means, of direct or indirect control of the whole or parts of one or more other undertakings”.

24 There are two alternative ways to reach turnover thresholds for EU dimension. The first alternative requires: (i) a combined worldwide turnover of all the merging firms over €5 000 million, and (ii) an EU-wide turnover for each of at least two of the firms over €250 million. The second alternative requires: (i) a worldwide turnover of all the merging firms over €2 500 million, and (ii) a combined turnover of all the merging firms over € 100 million in each of at least three Member States, (iii) a turnover of over €25 million for each of at least two of the firms in each of the three Member States included under ii, and (iv) EU-wide turnover of each of at least two firms of more than €100 million. In both alternatives, an EU dimension is not met if each of the firms archives more than two thirds of its EU-wide turnover within one and the same Member State.

25 EC, Case M.8228, *Facebook /Whatsapp*, 17.05.2017.

26 Commission Notice on Case Referral in respect of concentrations; OJ 2005/C 56, 5 March 2005, 2. This Notice is meant to “describe in a general way the rationale underlying the case referral system […], to catalogue the legal criteria that must be fulfilled in order for referrals to be possible, and to set out the factors which may be taken into consideration when referrals are decided upon. The Notice also provides practical guidance regarding the mechanics of the referral system, in particular regarding the pre-notification referral mechanism”—see para. 1.

27 Indeed, it reads that “The provisions of paragraph 1 may, however, be declared inapplicable in the case of: any agreement or category of agreements between undertakings, any decision or category of decisions by associations of undertakings, any concerted practice or category of concerted practices, which contributes to improving the production or distribution of goods or to promoting technical or economic progress, while allowing consumers a fair share of the resulting benefit, and which does not: (a) impose on the undertakings concerned restrictions which are not indispensable to the attainment of these objectives; (b) afford such undertakings the possibility of eliminating competition in respect of a substantial part of the products in question.”.

28 See Petit, N. (2013) Droit européen de la concurrence*,* 2013, 457–458; Craig, P. (2012) EU Administrative Law, Oxford University Press, 5; C. F. Sabel, C.F. and Zeitlin, J. (2008) Learning from Difference. E.L.J. 271—298.

29 Commission Notice on cooperation within the Network of Competition Authorities (Text with EEA relevance), OJ C 101, 27.4.2004, 43–53, para 1, hereinafter “Cooperation Notice 2004”.

30 See “Cooperation Notice 2004”, para 5–15.

31 ibid, para 8.

32 ibid, para 12–13.

33 ibid, para 15.

34 See “Joint Statement of the Council and the Commission on the Functioning of the Network of Competition Authorities” (2002) (Joint Statement), paras 6–7, where Member States also accepted that their enforcement systems differ but nonetheless mutually recognized the standards of each other’s system as a basis for cooperation.

35 Under Article 28 of Reg. 1/2003, the exchange of information described above must take into consideration issues such as: business secrecy and confidential information; the scope within which the evidence collected can be used; and the use of this information in relation to the cases where individuals violate EU competition law.

36 See, e.g., “Cooperation Notice 2004”, para 43, which reads, “[w]ithin the network of competition authorities the Commission, as the guardian of the Treaty, has the ultimate but not the sole responsibility for developing policy and safeguarding consistency when it comes to the application of EC competition law.”

37 It is worth noticing that the type of cooperation mentioned herein is part of a wider range of cooperation arrangements and techniques existent under the SSM. More specifically, while this paper focuses on the cooperation between national and European authorities (namely, the NCAs and the ECB) strictly from an institutional standpoint, cooperation also exists amongst other stakeholders (for example, in the interplay of international financial organizations and the ECB in the form of thematic working groups), as well as in terms of a more concrete operational approach, as seen in the JSTs (see footnote 13), and also in terms of exchange of information and data benchmarking (as done between the ECB and other European authorities), all of which are unarguably crucial forms of cooperation in the European model for financial supervision.

38 The wording is as follows: “With respect to the first subparagraph above, a credit institution or financial holding company or mixed financial holding company shall not be considered less significant, unless justified by particular circumstances to be specified in the methodology, if any of the following conditions is met.” Reference to the existence of “particular circumstances” is— actually, not very clearly— already set out in Article 6, para. 4 of the SSMR, but it is better articulated in Article 70 of the Framework Regulation. According to the latter, “particular circumstances, as referred to in the second and fifth subparagraphs of Article 6 (4) of the SSMR (hereinafter the ‘particular circumstances’) exist where there are specific and factual circumstances that make the classification of a supervised entity as significant inappropriate, taking into account the objectives and principles of the SSMR and, in particular, the need to ensure the consistent application of high supervisory standards.” Paragraph 2 of the same Article also specifies that the term “particular circumstances” must be interpreted restrictively: the derogation is, therefore, exceptional.

39 C-450/17 P, Landeskreditbank Baden-Württemberg—Förderbank v European Central Bank, ECLI:EU:C:2019:372. Most recently, on the case, see A. Riso, A Prime for the SSM before the Court: the L-Bank case (2021). In: C. Zilioli and K.-P. Wojcik (eds.) Judicial Review in the European Banking Union*,* Edward Elgar, pp. 494:504.

40 Lowe, P. (2004) The Role of the Commission in the Modernisation of EC Competition Law’, Speech at the UKAEL Conference, 6.

41 The Commission usually intervenes with reference to cases (a) presenting strong transnational aspects, i.e., involving more than 3 Member States; (b) which are closely linked to other European Union provisions that are exclusively or more effectively applied by the Commission; or (c) presenting new elements which would benefit from the adoption of a Commission decision in order to develop a common stance and ensuring effective consistent treatment of similar cases in the future.

42 For an overview on the implementation of Regulation 1/2003, see Commission, “Ten Years of Antitrust Enforcement under Regulation 1/2003: Achievements and Future Perspectives” COM(2014) 453.

43 Usually, the EC advocates a case in one of the following situations, i.e., when: (a) Network members envisage conflicting decisions in the same case; (b) Network members envisage a decision which is obviously in conflict with consolidated case law; (c) Network member(s) is (are) unduly drawing out proceedings; (d) there is a need to adopt a Commission decision to develop European competition policy; and (e) the national competition authority does not object.

44 In addition to Article 11 discussed in the text, Article 12 allows the NCAs to ask the Commission to provide them with information. In principle, the Commission should accede to such request unless there are compelling reasons to refuse. The request can refer to any kind of information: documents, statements, digital information. To be sure, apart from the case-related information exchanges, within the ECN there are also more general exchanges ranging from sectoral policy issues to a wide range of other issues. For example, also information about intended or submitted amicus curiae briefs to national courts can be exchanged on a voluntary basis in order not to duplicate efforts.

45 See Commission, “Ten Years of Antitrust Enforcement under Regulation 1/2003” SWD(2014) 230/2, 71, evidencing that the latter case occurs very rarely and, in any case, “such observations are necessarily advisory in nature and leave the responsibility of the authority dealing with the case entirely intact.”

46 Cooperation Notice 2004, para 54. It has to be taken into account that an additional letter (e) indicates that transfer of a case to the Commission at such late stage can occur only with the agreement of the national authority which is taking care of it. Nonetheless, the existence of such provision has been very much criticized, as it is not clear whether NCAs could actually oppose to a request in this sense from the Commission. In the absence of relevant case law, the question remains open.

47 According to SWD 2014, n 37 supra, 72, “to date, the Commission has not used the power to initiate proceedings after the reception of an envisaged decision pursuant to Article 11(4) over the entire period of application of Regulation 1/2003, essentially for the reason that case practice in the ECN has developed in a broadly coherent manner and more upstream means of interaction have been preferred as being more efficient.”

48 See Gortsos, C. V. (2015) The two main pillars of the European Banking Union: the legal framework in a ‘nutshell. In: Binder, J.-H. and Gortsos, C.V. (2015) Banking Union. A Compendium. C.H. Beck, p. 21, who argues “This mechanism is neither an authority nor an agency and has no legal personality. It is defined as meaning the ‘system of financial supervision’ composed of the ECB and the national competent supervisory authorities of participating Member States, including those of Member States with a derogation, if the latter have established a ‘close cooperation’ according to Article 7.”.

49 It should be noted that, after the introduction of the SSM, there were those who believed that the new system was based not on a logic of centralization, but on a model of decentralization of responsibilities. In the light of the *Landeskreditbank* decision, those statements today almost make us smile: see Bundesbank, *European Single Supervisory Mechanism for banks—a first step on the road to a banking union, Bundesbank Monthly Report*, July 2013, 16 et seq., available at https://www.bundesbank.de/resource/blob/707368/193457d491641f97b82a068837fd7e0e/mL/2013-076-single-data.pdf*;* Texeira, P.G. (2014) The Single Supervisory Mechanism: Legal and institutional foundations. In: Quaderni di Ricerca Giuridica della Consulenza Legale della Banca d'Italia, pp. 73-93; Figliolia, C. (2016) I rapporti con le banche centrali nazionali. In: Chiti P. and Santoro V. (eds), *L’unione bancaria europea*, specifically at p. 225. The attitude of the interpreters was at the time more oriented towards a more diversified description of the structures between the ECB and the NCAs, discarding, however, the thesis, perhaps more intuitive, of the classic scheme of the "delegation" of functions, see Wymeersch, W. (2012) The European Banking Union, a first analysis, Financial Law Institute Working Paper, 17 et seq.

50 See Macchia, M. (2016) Modelli di coordinamento della vigilanza bancaria. Riv. trim. dir. pubb. 367; with an emphasis on the integration profile, Angeloni, I. (2017) La BCE e le Autorità di vigilanza nazionali: cooperazione e sfide’ comuni*,* available at: https://www.bankingsupervision.europa.eu/press/speeches/date/2017/html/ssm.sp170922_1.it.html*.*

51 In these terms, Wymeersch, E. n 41 supra, 3 et seq.

52 See n 42 supra.

53 Magliari, A. (2015) I procedimenti amministrativi di vigilanza bancaria nel quadro del Single Supervisory Mechanism. Il caso dell’applicazione dei diritti nazionali da parte della BCE, *Riv. dir. banc.* July-Sept. 271:301.

54 Ferran, E. (2015) The Existential Search of the European Banking National, ECGI Working Paper, 297, at p., 3: "the ECB directly supervises the "significant" entities via joint supervisory teams, which includes staff from both NCAs and the ECB. Whilst the NCAs continue to be responsible for the day-to-day supervision of less significant entities, they do so under the oversight and control of the ECB. The SSM is supported by the Single Resolution Mechanism (SRM) which comprises a centralized decision-making body (Single Resolution Board or SRB) and fund (Single Resolution Fund or SRF).”.

55 D’Ambrosio, R. (2016) Meccanismo di vigilanza unico. In: *Enciclopedia del Diritto*, Milano, Annali IX, p. 594.

56 ibid.

57 Lamandini, M. (2015) Il diritto bancario dell’Unione. Banca borsa tit. cred*.* 423:457, with ample references to the theses of S. Cassese. On composite administrative procedures, see Brito, F., 'Judicial review of composite administrative procedures in the Single Supervisory Mechanism: Berlusconi'. Common Market Law Review, Volume 56, Issue 5 (2019), pp. 1355–1378 and also Brito, F., 'Derivative illegality in European composite administrative procedures', Common Market Law Review, Volume 55, Issue 1 (2018), pp. 101–134.

58 *Ex multis*, see Barbagallo, G. (2014) Il rapporto tra BCE e autorità nazionali nell’esercizio della vigilanza, speech at the Conference *Unione bancaria: istituti, poteri e impatti economici* held at Università LUISS, Rome, 26 February 2014, available at https://www.bancaditalia.it/pubblicazioni/interventi-vari/int-var-2014/Barbagallo_260214.pdf; F. Guarracino, *Il meccanismo unico di vigilanza sugli enti creditizi tra diritto primario e riforma dei Trattati*, (2013) 3 *Rivista trimestrale di diritto dell’economia* 171.

59 The need for uniformity is explicitly stated in Article 16 of Regulation 1/2003.

60 Directive (EU) 2019/1 of the European Parliament and of the Council of 11 December 2018 to empower the competition authorities of the Member States to be more effective enforcers and to ensure the proper functioning of the internal market, OJ L 11, 14.1.2019, 3–33.

61 It has been a matter for intense debate whether the ECB has the legitimacy to issue Regulations in the field of Prudential supervision. Originally, the issue arose because the SSM rules are not coordinated with the Single Rulebook and, in particular, with CRD IV and CRR. These legal acts leave however many options open, and several gaps that, depending on the subject matter, may be filled in by “Member States” or by “competent authorities.” Nonetheless, the debate on this point seems to have, at least for the moment, abated. For a general overview of the problems connected with the SSM, see: Masciandaro, D. and Nieto, M.J. (2012) Gouvernance du mécanisme de supervision unique: quelques réflexions. Revue d’économie financière 112:51-70. About the ECB’s institutional capacity and legal competence, in a critical sense, see for example: Kern, A. (2016) The European Central Bank and Banking Supervision: The Regulatory Limits of the Single Supervisory Mechanism, *ECFR* 13: 467-494.

62 Regulation (EU) 2018/1845 of the European Central Bank of 21 November 2018 on the exercise of the discretion under Article 178(2)(d) of Regulation (EU) No 575/2013 in relation to the threshold for assessing the materiality of credit obligations past due (ECB/2018/26), OJ L 299, 26.11.2018, 55–57.

63 Regulation (EU) 2015/534 of the European Central Bank of 17 March 2015 on reporting of supervisory financial information (ECB/2015/13), OJ L 86, 31.3.2015, 13–151.

64 OJ L 123, 27.4.2004, 18–24. See also Decision 2011/695/EU of the President of the European Commission of 13 October 2011 on the function and terms of reference of the hearing officer in certain competition proceedings (OJ L 275/29, 20.10.2011).

65 Antitrust law often operates in a layered framework, where legislative acts enacted by the Council are further complemented by Commission Regulations, which in turn are clarified by specific Guidelines, which restate the law and explain legislators' intentions. This is the case, for instance, (i) in relation to horizontal cooperation agreements, in addition to Council Regulation 2821/71/EEC, the Commission Regulation 1217/2010/EU of 14 December 2010 on the application of Article 101(3) TFEU to certain categories of research and development agreements, OJ L 335/36, 18.12..2010; the Commission Regulation 1218/2010/EU of 14 December 2010 on the application of Article 101(3) TFEU to certain categories of specialization agreements, OJ L 335/43, 18.12.2010, and the Guidelines on the applicability of Article 101 TFEU to horizontal cooperation agreements, OJ C 11/1, 14.1.2011; (ii) in connection to vertical agreements consider, beyond Council Regulation 19/65/EEC, the Commission Regulation 330/2010/EU of 20 April 2010 on the application of Article 101(3) TFEU to categories of vertical agreements and concerted practices, OJ L 102/1, 23.4.2010, and the Guidelines on Vertical Restraints, OJ C 130/1, 19.5.2010; and (iii) finally, as to Technology transfer agreements, consider Commission Regulation 772/2004/EU of 27 April 2004 on the application of Article 81(3) of the Treaty to categories of technology transfer agreements, OJ L 123/11, 27.4.2004 and the Guidelines on the application of Article 81 of the EC Treaty to technology transfer agreements, OJ C 101/2, 27.4.2004.

66 Case C-911/19 (Fédération bancaire française v Autorité de contrôle prudentiel et de résolution) [ECLI:EU:C:2021:599]. For a complete analysis of the decision, see Annunziata, Filippo, 'The Remains of the Day: EU Financial Agencies, Soft Law and the Relics of Meroni' European Banking Institute Working Paper Series 2021—no. 106, 2021.

67 See Bax, R. and Witte, A. (2019) The taxonomy of ECB instruments available for banking supervision, published as part of the ECB Economic Bulletin, Issue 6/2019, and available at https://www.ecb.europa.eu/pub/economic-bulletin/articles/2019.

68 Case C-911/19 (Fédération bancaire française v Autorité de contrôle prudentiel et de résolution) [ECLI:EU:C:2021:599]. The controversy leading to the Court’s decision of July 15 2021 revolves around the Guidelines on product oversight and governance for banking retail products, issued by the European Banking Authority ("EBA") in 2016 (hereinafter also the “2016 Guidelines”) 48. Following the “comply or explain” mechanism that is proper to the ESAs’ Guidelines, the French ACPR informed, in September 2017, throughout a Notice, that it would comply with the EBA’s Guidelines. The French Banking Federation (FBF) challenged the Notice published in this respect by the ACPR before the French Conseil d’Etat, arguing that the Guidelines had been issued by the EBA exceeding its powers and competences. For a complete analysis of the decision, see Annunziata, Filippo, 'The Remains of the Day: EU Financial Agencies, Soft Law and the Relics of Meroni' European Banking Institute Working Paper Series 2021—no. 106, 2021.

69 See, e.g., Commission Notice on the best practices for the conduct of proceedings concerning Articles 101 and 102 TFEU, OJ C 308/6, 20.10.2011; Commission Notice on cooperation within the Network of Competition Authorities, 2004/C 101/03, OJ C 101/43, 27.4.2004; Commission Notice on the co-operation between the Commission and the courts of the EU Member States in the application of Articles 81 and 82 EC, OJ C 101/54, 27.4.2004; Guidelines on the method of setting fines imposed pursuant to Article 23(2)(a) of Regulation 1/2003/EC, OJ C 210/2, 1.9.2006; Commission Notice on the handling of complaints by the Commission under Articles 81 and 82 of the EC Treaty, OJ C 101/65, 27.4.2004; Commission Notice on informal guidance relating to novel questions concerning Articles 81 and 82 of the EC Treaty that arise in individual cases (guidance letters), OJ C 101/78, 27.4.2004; Commission Notice on the rules for access to the Commission file in cases pursuant to Articles 81 and 82 of the EC Treaty, Articles 53, 54 and 57 of the EEA Agreement and Council Regulation (EC) No 139/2004, OJ C 325/7, 22.12.2005); Commission Notice on the conduct of settlement procedures in view of the adoption of Decisions pursuant to Article 7 and Article 23 of Council Regulation 1/2003/EC in cartel cases, OJ C 167/1, 2.7.2008; Commission Notice on Immunity from fines and reduction of fines in cartel cases, OJ C 298/17, 8.12.2006.

70 Commission Notice on the definition of relevant market for the purposes of Community competition law, OJ C 372, 9.12.1997.

71 Commission Notice on agreements of minor importance which do not appreciably restrict competition under Article 81(1) of the Treaty establishing the European Community (de minimis), OJ C 368/13, 22.12.2001.

72 Commission Notice: Guidelines on the application of Article 81(3) of the Treaty, OJ C 101/97, 27.4.2004.

73 Guidelines on the effect on trade concept contained in Articles 81 and 82 of the Treaty, OJ C 101/81, 27.4.2004.

74 Commission Consolidated Jurisdictional Notice under Council Regulation (EC) No 139/2004 on the control of concentrations between undertakings, OJ C95/1, 16.04.2008.

75 C-526/14, Tadej Kotnik and Others v Državni zbor Republike Slovenije, ECLI:EU:C:2016:570.

76 For the sake of clarity, it is to be noted that the Commission cannot set enforcement priorities of NCAs. Guidance on the Commission's enforcement priorities in applying Article 82 of the EC Treaty to abusive exclusionary conduct by dominant undertakings, OJ C 45/7, 24.2.2009.

77 *Antitrust: Joint statement by the European Competition Network (ECN) on application of competition law during the Corona crisis*, available at https://ec.europa.eu/competition/ecn/202003_joint-statement_ecn_corona-crisis.pdf and Communication from the Commission, *Temporary Framework for assessing antitrust issues related to business cooperation in response to situations of urgency stemming from the current COVID-19 outbreak*, CI 116/7, 20.4.2020.

78 ibid.

79 In the 2009 Guidance the Commission clearly stated that exclusionary and anticompetitive abuses would have been its prior target. Nevertheless, since then, the EC and Competition NCAs have prosecuted several cases about exploitative and discriminatory abuses.

80 See, in relation to the working of the ECN, Article 20 and 22 of Reg. 1/2003.

81 See lately on these issues Markakis, M. (2021) Accountability in the Economic and Monetary Union, Oxford University Press.

82 See, for example, T-346/94 France Aviation; T-290/97 Mehibas Dordtselaan; C-64/05, *Sweden v Commission*, ECLI:EU:C:2007:802.

83 Judgment of the Court (Fifth Chamber) of 3 December 1992, *Oleificio Borelli SpA v Commission of the European Communities*: “[i]n an action brought under Article 173 of the Treaty the Court has no jurisdiction to rule on the lawfulness of a measure adopted by a national authority.” (10) That position cannot be altered by the fact that the measure in question forms part of a Community decision-making procedure. (…) (12) In those circumstances, any irregularity that might affect the opinion *[of the national authorities forming part of a Community decision-making process]* cannot affect the validity of the decision by which the Commission refused the aid applied for. (13) Accordingly, it is for the national Courts, where appropriate after obtaining a preliminary ruling from the Court, to rule on the lawfulness of the national measure at issue.”

84 Case C-219/17, Silvio Berlusconi and Finanziaria d'investimento Fininvest SpA (Fininvest) v Banca d'Italia and Istituto per la Vigilanza Sulle Assicurazioni (IVASS), request for a preliminary ruling from the Consiglio di Stato, ECLI:EU:C:2018:1023. On the decision, see Buono, G. (2021) Banking authorizations and the acquisition of qualifying holdings as unitary and composed procedures and their judicial review. In: Zilioli C. and Wojcik, K.-P. (eds.) Judicial Review in the European Banking Union Edwar Elgar, pp. 251:285.

85 Judgment of the Court of 15 January 1987, *Krohn & Co. Import-Export GmbH & Co. KG v Commission of the European Communities*. For a wide assessment of the division between the judiciaries of the EU and of the Member States in the context of the Banking Union, see Lehmann, M. Jurisdiction, locus standi and the circulation of judgments in the Banking Union. In: Zilioli C. and Wojcik, K.-P. (eds.) Judicial Review in the European Banking Union, Edward Elgar, pp. 77:97.

86 As also clarified and developed by the landmark CJEU decision on Landeskreditbank, judicial review in the context of the SSM is also composed of an additional element: the Administrative Board of Review ("ABoR"), which carries out a final internal review of the ECB's supervisory decisions and serves as a cushion in-between the administrative and judicial spheres, see e.g. Brescia Morra, C., Smits, R. & Magliari, A. 'The Administrative Board of Review of the European Central Bank: Experience After 2 Years'. Eur Bus Org Law Rev 18, 567-589 (2017).

87 The ECB—similarly to most Supervisors in the EU—has the power to impose administrative penalties on supervised entities. These may be applied in case of breach of regulations under directly applicable EU banking law, or breach of ECB legal acts. With regard to other cases of breaches of EU banking law (including breach of national law, implementing EU Directive), forms of cooperation between the ECB and National authorities have been identified. The topic is governed by Article 18 of the SSMR and Article 120-137 of the ECB Framework Regulation (Part X). Within this context, on 16 April 2014 the Governing Council of the ECB adopted the ECB Regulation (EU) No 469/2014, and a Recommendation "for a Council Regulation amending Council Regulation (EC) No 2532/98 concerning the powers of the European Central Bank to impose sanctions" (ECB/2014/19). Council Regulation ((EU) 2015/159) was adopted on 27 January 2015: its purpose has been to adapt the EU legal framework on ECB sanction-imposing powers to the functioning of the SSM. In particular, the amendments aim at clarifying that its provisions do not apply to the sanctions that may be imposed by the ECB in the exercise of its supervisory tasks, since the latter are covered exclusively by Article 18 of the SSMR. On the other hand, Council Regulation (EU) 2015/159 aims at bringing Council Regulation (EC) No 2532/98 in line with Article 18 of the SSMR, in particular with regard to topics such as: (i) the upper limits of sanctions imposed by the ECB in the exercise of its supervisory task; (ii) identification of specific procedural rules for sanctions imposed by the ECB in the exercise of its supervisory tasks; (iii) specific time-limits for administrative penalties imposed by the ECB in the exercise of its supervisory tasks (new Article 4a-4c). On the case law of the CJEU in relation to the ECB decisions imposing pecuniary penalties, see Wissink, L. (2021) The VQ case T-203/18: administrative penalties by the ECB under judicial scrutiny, and Poscia, J. (2021) The VTB case: administrative penalties and administrative measures, both in Zilioli C. and Wojcik, K.-P. (eds.) Judicial Review in the European Banking Union, Edward Elgar, at 542:551 and 571:579.

88 See ECtHR, Engels and others v. the Netherlands (App. 5100/71; 5101/71; 5102/71; 5354/72; 5370/72). According to this decision, there are three criteria for the applicability of the criminal aspect of Article 6 HRCo: 1) the domestic classification; 2) the nature of offense; 3) the severity of the potential penalty. The last criteria seem to have a strong significance in the matter we are studying.

89 This can be seen in practice, for instance, in cases of undertakings that have allegedly entered an anticompetitive exclusive distribution agreement. The undertakings at stake, to avoid arguing the case, can volunteer to modify the agreement to make it procompetitive.

